# Functions and Regulatory Mechanisms of bHLH Transcription Factors during the Responses to Biotic and Abiotic Stresses in Woody Plants

**DOI:** 10.3390/plants13162315

**Published:** 2024-08-20

**Authors:** Tengyue Yan, Xiaochun Shu, Chuanli Ning, Yuhang Li, Zhong Wang, Tao Wang, Weibing Zhuang

**Affiliations:** 1 Jiangsu Key Laboratory for the Research and Utilization of Plant Resources, Institute of Botany, Jiangsu Province and Chinese Academy of Sciences (Nanjing Botanical Garden Memorial Sun Yat-Sen), Nanjing 210014, China; ytydyx2022@163.com (T.Y.);; 2 Yantai Agricultural Technology Extension Center, Yantai 264001, China

**Keywords:** bHLH transcription factors, stress tolerance mechanism, woody plants, biotic stress response, abiotic stress response

## Abstract

Environmental stresses, including abiotic and biotic stresses, have complex and diverse effects on the growth and development of woody plants, which have become a matter of contention due to concerns about the outcomes of climate change on plant resources, genetic diversity, and world food safety. Plant basic helix–loop–helix (bHLH) transcription factors (TFs) are involved in a variety of physiological processes and play an important role in biotic and abiotic stress responses of woody plants. In recent years, an increasing body of studies have been conducted on the bHLH TFs in woody plants, and the roles of bHLH TFs in response to various stresses are increasingly clear and precise. Therefore, it is necessary to conduct a systematic and comprehensive review of the progress of the research of woody plants. In this review, the structural characteristics, research history and roles in the plant growth process of bHLH TFs are summarized, the gene families of bHLH TFs in woody plants are summarized, and the roles of bHLH TFs in biotic and abiotic stresses in woody plants are highlighted. Numerous studies mentioned in this review have shown that bHLH transcription factors play a crucial role in the response of woody plants to biotic and abiotic stresses. This review serves as a reference for further studies about enhancing the stress resistance and breeding of woody plants. Also, the future possible research directions of bHLH TFs in response to various stresses in woody plants will be discussed.

## 1. Introduction

Various biotic and abiotic stresses in the environment serve as the primary factors that inhibit nearly all the physiological and biochemical processes [1], resulting in consequences such as decreased crop yields [2], loss of medicinal components [3], slow growth of wood [4], and other adverse outcomes. Increasingly, environmental challenges such as extreme temperatures, high salinity, drought, and heavy metal contamination can seriously affect plant growth [5]. Trees occupy a large area of land on the Earth and play a role in many aspects of human life [6]. Therefore, it is necessary to explore the stress response mechanisms, pay attention to their condition and improve stress tolerance of woody plants.

A series of gene expression regulation [7], accumulation of metabolites [8], and signal transduction [9] occurred in the evolutionary development of woody plants in response to various stress environments, and many unique phenotypes were gradually formed [10]. To better understand the woody plants’ mechanisms to combat various stresses and improve stress tolerance in woody plants, an increased number of studies have been carried out in the field of plant stress response at the molecular level [11,12]. It has been confirmed that in order to adapt to harsh environmental stresses, woody plants have successfully evolved complex responding mechanisms in the long process of evolution, in which TFs (like MYB TFs, bHLH TFs, and WD40 TFs) play an important role in the regulation of gene expression in response to environmental factors [7,13,14,15]. 

Transcription factors are a class of proteins that bind exclusively to specific sequences at the 5′ end in a gene’s upstream region to ensure that the target gene is expressed with a specific intensity at a specific time and location [16]. These transcription factors can regulate the expression of genes related to growth and development in plants at the transcriptional level and then regulate the growth processes, such as the release and decomposition of growth hormones [17], the absorption of trace elements [18], and the regulation of secondary metabolites [19]. TFs play a crucial role in various physiological responses of plants under various stresses [20]. At present, many scientists have focused on identifying and characterizing the roles of various TFs in woody plants under various stresses, and many achievements have been obtained in the stress resistance studies in woody plants.

bHLH TFs, one of the largest families of TFs in plants [21], is classified based on its typical basic region and helix–loop–helix domain. By specifically binding to cis-acting elements in related gene promoter regions, bHLH TFs regulate the transcription of target genes, thereby affecting the adaptive responses to biotic and abiotic stresses [22], the growth [23], and the secondary metabolic networks in woody plants [24,25].

Over the past decades, bHLH TFs and their molecular mechanisms in response to various stresses have been extensively characterized and studied. Previous studies on bHLH TFs mainly focused on some model plants, such as tobacco [26,27] and *Arabidopsis* [28,29,30]. Thereafter, the study field had expanded to crops [31,32,33]. In recent years, an increasing number of experiments have been conducted to study the stress response mechanisms of bHLH TFs in woody plants. However, there are few systematic and general reviews of research progress in woody plants. This review is mainly focused on the function and mechanisms of bHLH TFs. The structural characteristics, research history, and roles in the plant growth process of bHLH TFs were also summarized. In addition, the molecular mechanisms in response to various stresses in woody plants were also explained. This review provides a reference for future application of bHLH TFs in the improvement of stress resistance and the breeding of woody plants and a reference for future studies on the molecular mechanism of interaction between bHLH TFs and other TFs. 

## 2. Structure of bHLH TFs

**Figure 1:**
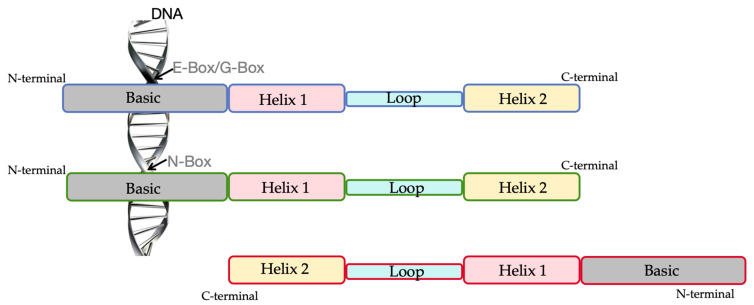
The structural representation of bHLH TFs. There are three bHLH TFS in the diagram: one binds to the E-Box/G-Box in the DNA sequence; one binds to the N-Box in the DNA sequence; and one does not bind to DNA sequence.

As one of the largest TF families, the bHLH TFs family is widely distributed in plants. The bHLH TFs usually contain approximately 60 amino acids and conservatively include two functionally distinct regions, the typical basic region and helix–loop–helix domain [34] (Figure 1). The HLH region is composed of hydrophobic amino acids, of which Isoleucine (I), Leucine (L), and Valine (V) is necessary for dimerization, and they stabilize the structure of the dimer. bHLH TFs have the function of taking part in protein-protein interaction and regulating the expression of the target gene. The N-terminal domain, responsible for DNA binding, contains about 15 to 20 amino acids, including basic amino acid residues. Among these amino acid sequences, including basic amino acids, there are conserved amino acids responsible for the recognition of the DNA cis-element E-Box (5′-CANNTG-3′) and/or G-Box (5′-CACGTG-3′) in the promoter sequences of target genes (Figure 1). Outside the bHLH domain, bHLH TFs usually show low or no sequence conservation [35]. Nevertheless, some functionally and/or evolutionary-related bHLH proteins may share additional motifs and the members in the same subfamily classified on this basis contain similar motifs [36].

## 3. Research History of bHLH TFs

The bHLH motif was first identified by Murre et al. in 1989 [37]. The existence of the HLH motif was confirmed by the three-dimensional structure of the basic HLH (bHLH)-leucine zipper (LZ) factor Max [38,39]. Subsequently, the domain of bHLH TF that can bind with DNA was identified. The three-dimensional structure of the bHLH/leucine zipper domain of the TF Max complexed with DNA was determined by X-ray crystallography at 2.9 Å resolution [40]. After the discovery of the bHLH motif in murine [37], the presence of bHLH superfamily in plants was first identified in maize [41]. Since then, more studies about bHLH TFs have been carried out in plants, and there are more studies focused on the genome-wide analyses of the *bHLH* gene families in different species [42,43]. Further studies have attempted to divide the *bHLH* gene families into smaller and more specific subfamilies based on sequence homology [44]. Different numbers of the *bHLH* gene families have been identified in different species through genome-wide identification. A total of 133 *AtbHLH* genes have been identified in *Arabidopsis*, which were rearranged into 12 subgroups according to their genetic structure, number of introns, and conservative motifs [45]. In addition, 100 *NtbHLH* genes in the *Nicotiana tabacum* genome were split into 15 major groups based on the conserved domains and their phylogenetic relationships using Hidden Markov Model profiles [46]. 

As research deepens, the identification of *bHLH* gene families is carried out not only in model plants, but also in many crops. For example, 187 *bHLH* genes of *Setaria Italica* (SibHLH TFs) were divided into 21 subfamilies and two other orphan genes based on the number of conserved domains and their different structures [31]. A total of 122 *bHLH* genes were identified in pepper, which were categorized into 21 subfamilies based on the specific conserved amino acids of TFs and the imparity of other conserved structural domains [47]. With the deepening of research and the advancement of technology, the exploration of *bHLH* gene families in woody plants has begun. A total of 104 *bHLH* genes in *Prunus sibirica* have been identified, which were classified into 23 subfamilies and unevenly distributed on eight chromosomes [48]. Based on the whole-genome sequence data of *Cyclocarya paliurus*, 159 *bHLH* genes were successfully identified and classified into 26 subfamilies [49]. According to the similarities of sequences and phylogenetic relationships, 136 *bHLH* genes in yellow horn (*Xanthoceras sorbifolia Bunge*) have been identified and divided into 26 subfamilies [50]. A total of 206 *bHLH* genes from *Sweet osmanthus* (*Osmanthus fragrans*) were classified into 25 subfamilies [51]. Due to their evolutionary relationships, 100 *bHLH* genes were identified and classified into 21 subfamilies in *Prunus mume* [52]. A total of 85 *GbbHLH* genes from *Ginkgo biloba* were classified into 17 subfamilies based on their phylogenetic analysis [53]. Through phylogenetic analysis, 167 *bHLH* genes in *Populus tremula* have been identified and divided into 15 subfamilies [54]. Based on the sequence similarities and phylogenetic relationships, 185 *PdbHLH* genes were classified into 15 groups in the *Populus deltoids* [55]. In *Rosa persica*, 142 *RbebHLH* genes have been identified and divided into 21 subfamilies by their similar structures and motifs [56]. The summary of these bHLH TFs mentioned above is shown in Supplemental Table S1. In conclusion, the identifications of bHLH TF families in different species provide a reliable base for future studies of their structural characteristics and functions in woody plants.

## 4. Roles of bHLH TFs in Plant Growth

The functions of many members of the bHLH family have been explored in different plants. In plants, there are great differences in the functional roles of the bHLH TFs, and the biological functions generated by their respective actions on target genes also vary greatly due to the numerous members of the *bHLH* gene family. Various physiological changes in plants are closely related to their transcriptional regulatory networks. bHLH TFs are involved in many growth and development processes, including the germination of seed [57], the development of flower organs [58], the formation of root systems [59], the ripening of fruit [60] and the branching of shoots [61]. Also, bHLH TFs are related to the flowering process. For example, PtbHLH TFs are involved in the flowering process in *Populus trichocarpa* [62], and the ectopic expression of a poplar *bHLH* gene (*PtbHLH173*) could contribute to early flowering in *Arabidopsis* [63]. In jujube trees, ZjbHLH TFs perform different functions during the development of flowers and fruit [64]. In addition, bHLH TFs are also involved in the secondary metabolic network of plants. It has been well established that bHLH TFs control the transcriptional regulation of flavonoid biosynthesis (e.g., the anthocyanin biosynthetic pathway) in many woody plants, and *bHLH* genes involved in the anthocyanin biosynthesis primarily belong to subfamily IIIf. PdTT8, a bHLH protein from *Populus deltoids*, can activate the expression of genes associated with anthocyanin biosynthesis through its direct interaction with PdMYB118 [25]. Terpenoids can protect plants from environmental damage (e.g., ultraviolet light, pests, diseases, and bacteria) and play a role in the defensive mechanisms in response to abiotic stresses [65,66]. For example, LcbHLH78 is involved in the production of terpenoids in *Litsea cubeba*, and the transient overexpression of *LcbHLH78* enhanced the accumulation of terpenoids (linalool, citronellal, geraniol etc.) in *Litsea cubeba* leaves [67]. In addition, bHLH TFs also regulate plant growth by participating in light signaling. PIF7, a bHLH TF in *Arabidopsis*, is a phytochrome interacting factor (PIF), which could participate in seedling de-etiolation [68]. The above studies laid a good foundation for further understanding of the roles of bHLH TFs in plants. However, it should be clear that the current research on bHLH TFs has mainly focused on the identification and expression analysis of family members, and the physiological effects and regulatory functions of most of them need to be systematically studied further.

## 5. Molecular Regulatory Mechanisms of Woody Plants Response to Various Stresses

**Figure 2:**
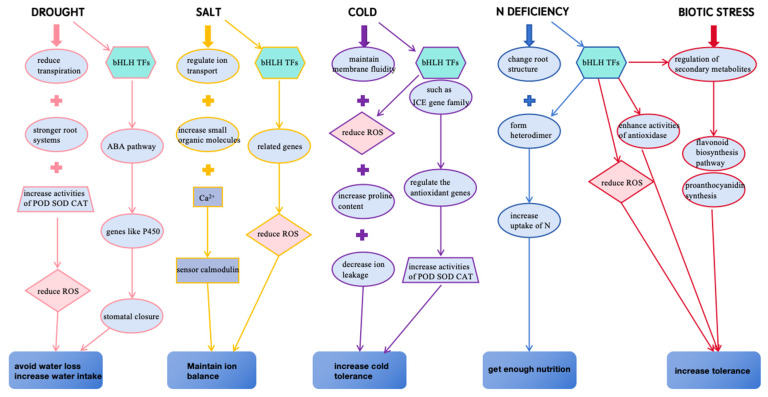
Schematic diagram of molecular stress response mechanisms under various stresses based on information in different woody plants. ROS—reactive oxygen species.

Various stresses in the environment can affect the normal physiological processes and basic metabolic pathways in woody plants and thus negatively impact their growth and development. To cope with stresses and keep normal growth under adverse environmental conditions, woody plants have evolved several strategies, which include both the physiological and molecular levels. The network of these mechanisms in response to stresses in woody plants is involved in the prevention or reduction of cell damage, the re-establishment of homeostasis and the recovery of growth (Figure 2).

In general, woody plants have multiple strategies to protect them from damage when they suffer from various stresses. When woody plants are under stress, the growth rate of woody plants is slowed down, the reproductive cycle is prolonged, and the energy needs to be reallocated in a way that allows for stress adaption but also maintains slow growth and reproduction [69,70]. Woody plants respond to various stresses at roughly two levels, including structural level and functional level [71]. At the structural level, the direction and growth speed of the root and stem are changed, and the structure of the cell membrane could change to prioritize the stability of the entry and exit of substances and the distribution and the opening and closing of stomata on the leaves are also affected in woody plants under various stresses [72]. At the functional level, several different mechanisms are involved in the responses of various stresses in woody plants. For example, the anabolism in cells decreases while the catabolism increases (such as the breakdown of proteins and starches) in woody plants under various stresses [73]. Woody plants produce defensive compounds such as sugars, protective proteins, and secondary metabolites (such as flavonoids) to optimize plant survival [74,75]. In addition, the tolerance of various stresses in woody plants is related to hormonal signaling, such as abscisic acid (ABA), jasmonic acid (JA), and salicylic acid (SA) pathways [76,77]. Besides, woody plants also evolved two specific systems to reduce the increase of reactive oxygen species (ROS) and the detrimental injuries caused by various stresses, including the enzymatic antioxidant system and the nonenzymatic compounds system [78]. There are several enzymes involved in the enzymatic antioxidant system such as peroxidase, glutathione reductase (GR), catalase (CAT), peroxidase (POD), and superoxide dismutase (SOD) [79]. A wide variety of nonenzymatic compounds such as proline [80,81], glutathione (GSH) [82], carotenoids [78], and ascorbic acid (Vitamin C) [83] are involved in the mechanisms of stress tolerance in woody plants. Stress tolerance mechanisms in woody plants are also involved with ion equilibrium (Ca^2+^, K^+^, and Na^+^) and other small metabolites like RNA and small peptides (e.g., cyclic dipeptides) [84,85,86,87]. In addition, hormone receptors and ion channels on the biological membrane and electrical signals are also involved [88]. Together, these different mechanisms of regulation play important roles in the stress responses of woody plants rather than acting separately and therefore help woody plants to establish the ability to resist various stresses.

Woody plants have evolved various strategies to cope with different stresses (Figure 2). In drought stress, woody plants protect themselves from damage by reducing water loss and increasing water intake. At the structural level, woody plants reduce water loss by closing their stomata to reduce transpiration [89], changing leaf characteristics [90] and gaining more water by stronger root systems [91]. At the functional level, woody plants have been reported to participate in the responses of drought stress and salt stress through ABA-mediated signaling pathways to induce stomatal closure [92]. Stress responses caused by low and high temperatures have a direct impact on the structure of molecules (DNA, lipids) and macromolecules (proteins, chromosomes) within cells [93]. Low temperature is a major stress for plants, especially woody plants. Increased tolerance under low-temperature stress is usually associated with reduced damage. Trees maintain the ability to grow under low-temperature stress through some mechanisms that promote frost resistance. For example, changing the saturation level of fatty acid chains in membrane lipids allows cells to maintain membrane fluidity at low temperatures [94,95]. Also, different tissues show different tolerances for cold resistance. For example, cells in bark tissues have higher resistance to cold stress than those in stems [96]. Beyond that, one common way for woody plants to respond to cold stress is to produce secondary metabolites, especially polyphenols, flavonoids, and terpenoids. For example, the anthocyanin content of blood orange fruits increased sharply when they were exposed to low temperatures [97]. The molecular mechanisms of woody plant response to salt stress are similar with these in response to drought stress. Previous studies have shown that salt stress causes a significant increase of Ca^2+^ sensor calmodulin in poplar leaves. This mechanism of signal transduction is most evident in salt-tolerant *populus euphratica*, which are more sensitive to changes of soil salinity than other poplar species [98]. Studies have shown that genes related to ion balance and osmotic homeostasis are up-regulated, and the absorption of Na^+^ and Cl^-^ is enhanced when the *populus euphratica* were under salt stress [99]. Woody plants frequently improve their tolerance by promoting the biosynthesis of some secondary metabolites when they are suffering from biotic stress. For example, poplar enhanced the resistance to *Septotis populiperda* by improving the biosynthesis of lignin [100]. In response to the lack of nutrients in the environment, woody plants usually increase the uptake of the deficient elements by changing the root structure. For example, *Populus* improve the absorption of nitrogen (N) by increasing the length and surface area of fine root [101]. The ability of woody plants in response to global environmental changes is critical to the future of the planet and humanity. An in-depth understanding of the molecular regulatory networks of woody plants under stress is still a major research topic in the future.

## 6. bHLH TFs in Response to Various Stresses in Woody Plants

In addition to their roles in the various basic physiological activities of plants, bHLH TFs are also involved in the responses to biotic and abiotic stresses in many woody plants. In recent years, environmental problems have seriously restricted the development of forestry. The analysis of the main stresses faced by the cultivation and growth of woody plants has become more important in the development of forestry ecological resources. Exploring the molecular mechanisms of the bHLH TFs’ response to various stresses in woody plants can not only explain the biological characteristics of woody plants but also clarify the stress network involved in the bHLH TFs in woody plants, which further provides directions for understanding the stress physiology of woody plants associated with bHLH TFs. Table 1 shows various bHLH TFs involved in different stress responses in woody plants.

### 6.1. Roles of bHLH TFs in Biotic Stress

There are always some “stumbling blocks” during the growth and development of woody plants, such as pathogens, microorganisms, insects, animals, and other organisms that constitute biotic stress to plants. Bacteria and fungi are very harmful to woody plants, resulting in poor tree growth, reduced fruit rate, late germination and flowering, early defoliation, and even the death of trees in severe cases. In recent years, an increasing number of studies have been devoted to exploring the response mechanisms of bHLH TFs to biotic stresses in woody plants. The stress resistance ensured by bHLH TFs in woody plants is generally related to the regulation of secondary metabolites. For example, transgenic poplar overexpressing *PalbHLH1* observably improved the resistance to *Botrytis cinerea* and *Dothiorella gregaria* infection through the higher accumulation of anthocyanin [132]. In sweet cherry fruits, the expression levels of 10 *bHLH* genes have been altered upon *Hop Stunt Viroid* infection, which indicated that these 10 *bHLH* genes might play important roles in the expression of the plant defense transcriptome [133]. Other secondary metabolites also play a broad role in the plant’s immune response to pathogens [135,136]. In walnut trees, the expression of genes involved in flavonoid and phenylpropanoid biosynthesis are regulated by bHLH TFs, and the accumulation of these secondary metabolites may lead to an enhancement of the tolerance to *Xanthomonas arboricola* pv. *juglandis* infection [134]. Furthermore, bHLH TFs can form highly dynamic complexes with other TFs (MYBs and WD40s) to respond various stresses [20]. For example, poplar leaves can increase the accumulation of proanthocyanins through the regulation of MYB–bHLH–WD40 complex when they suffer from the biotrophic rust fungi *Melampsora larici-populina* infection, which can be a shield for plants to protect themselves [137]. To cope with various biotic stresses, bHLH TFs in some woody plants can increase the expression of some genes involved in detoxification at the transcriptional level, thereby reducing the accumulation of harmful metabolites. For example, PalbHLH1 in poplar increased the activities of antioxidant enzymes and contributed to the release of H_2_O_2_, which enhanced the resistance to *Botrytis cinerea* and *Dothiorella gregaria* [132]. So far, there are still few studies on the mechanisms of bHLH to respond to the biotic stresses in woody plants. Future research directions may focus on the specific roles and regulatory pathways of bHLH TFs in the molecular mechanisms of woody plant responses to insects and bacterial and fungal diseases.

### 6.2. Roles of bHLH TFs in Woody Plants under Abiotic Stresses

#### 6.2.1. bHLH TFs Response to Drought Stress in Woody Plants

Globally, drought is a major environmental factor that severely limits plant yield and quality [138]. Closely related to human life and production, woody plants have always received significant attention, and there are many studies about bHLH TFs devoted to studying the stress tolerance mechanism in woody plants and ultimately improving their drought stress resistance. MdPIF3, a bHLH TF belonging to phytochrome interacting factors (PIFs) in apple trees (*Malus domestica*), played a positive role in response to drought stress [102]. In *Tamarix hispida*, the overexpression of *ThbHLH1* markedly improves the tolerance to drought stress by decreasing the accumulation of ROS [79]. HhbHLH2 has been reported to participate in the regulation of the drought stress response of *Hibiscus hamabo*, and the overexpression of *HhbHLH2* enhances the tolerance of drought stress in *Arabidopsis* [107]. It has been found that the enhancement of drought resistance caused by bHLH TFs in woody plants is generally related to signal transduction of ABA pathways, removement of ROS, and the equilibrium of ions. For example, MdCIB1 improved drought stress resistance of apple, and the ectopic expression of which in *Arabidopsis* also increases their drought tolerance through improving the activities of POD, SOD, and CAT, enhancing the sensitivity to ABA, and reducing water loss through stomatal closure induced by ABA [103]. MdSAT1 is a bHLHm1 TF in apple trees, which is homologous to Glycine max bHLH membrane 1 (bHLHm1). The overexpression of *MdSAT1* in apple calli resulted in a phenotype of increased tolerance to drought, and the overexpression of *MdSAT1* in *Arabidopsis* activated target genes in ABA pathway like the *P450* gene, improving the tolerance of drought [104]. Furthermore, MdbHLH130 from apple (*Malus domestica*) acts as a positive regulator of drought resistance in apple trees, and the overexpression of which increased tolerance of drought in apple trees by improving the activity of genes related to ROS-scavenging, and reduced the sensitivity of drought in apple trees by modulating the closure of stomatal [92]. PebHLH35 from *Populus euphratica* can activate the expression of *PeGSTU58* by directly binding to its promoter, thus improving drought tolerance by maintaining ROS homeostasis [105]. PxbHLH02 increases the activity of ROS scavenging in *Populus (Populus simonii × P. nigra)*, which can further promote their drought tolerance [106]. The transgenic *Arabidopsis* plant overexpressing *MrbHLH10* maintained higher ascorbate peroxidase (APX) activity and biomass accumulation under drought stress, while the RNAi plants had elevated susceptibility to drought stress, indicating that *MrbHLH10* mitigates abiotic stresses through the modulation of ROS scavenging [108]. For some woody plants, bHLH TFs apply more than one regulatory mechanism to enhance drought resistance. For example, PtrbHLH66 can improve drought resistance at both the structural level (the change of root growth) and the functional level (reduced accumulation of ROS) [109]. bHLH TFs function in certain stages of drought stress in woody plants rather than throughout the whole process. MabHLH144-like, a drought stress-responsive bHLH TFs in mulberry (*Morus alba* L.) are significantly upregulated at the early stage of drought stress [110]. Moreover, bHLH TFs improve plant drought resistance by regulating the expression of other genes associated with growth and development, which is also a major research direction at present. For example, the process of flowering in citrus is positively regulated by CibHLH96, which can bind to the promoter of *CiFD* to activate its expression and therefore could avoid more drought damage by producing seeds earlier to shorten their life cycle [111]. *SlbHLH* was highly expressed at 4% relative water content in *S. lepidophylla*, and overexpression of *SlbHLH_opt_* gene in *Arabidopsis* not only significantly increased plant growth, development, and integrated water-use efficiency but also significantly increased seed germination and green cotyledon emergence rates under water-deficit stress condition [112]. As mentioned above, the overexpression of *MrbHLHp10* from Chinese bayberry in transgenic *Arabidopsis* plants maintained higher activity of *ascorbate peroxidase* (*APX*) gene, indicating that *MrbHLHp10* might play a positive role in drought stress [108]. Above all, the regulatory mechanisms of bHLH TFs response to drought stress in woody plants need to be further explored.

#### 6.2.2. bHLH TFs Response to Salt Stress in Woody Plants

At present, soil salinization has become a global problem, and the salinization area is large and wide [139,140]. Exploring the stress responses of woody plants on high salt and alkali content can better understand the molecular mechanism of salt resistance in woody plants. As an important member of TFs, bHLH TFs play a crucial part in the responses to salt stress in woody plants, and usually improve salt tolerance by affecting the osmotic balance of woody plants. Plants usually adopt two osmotic regulation strategies in response to high-salt environments. One is to regulate ion transport; the second is to regulate the synthesis and accumulation of small organic molecules such as proline, ascorbic acid, flavonoids, and anthocyanin. *PsPRE1* is a bHLH TF from *Populus simonii* ‘Tongliao1’, and the overexpression of which in 84K poplar (*Populus alba × Populus glandulosa*) improved their salt tolerance due to the developed root system and higher CAT activity [113]. MdSAT1 is a bHLHm1 TF in apple, and the overexpression of which in *Arabidopsis* and apple calli contributes to their salt tolerance by modulating the expression of stress-related genes, such as *SOS* (*salt overly sensitive*) and *PLC5* (*phospholipase C*) [104]. *SlbHLH_opt_* gene is a codon-optimized form of the SlbHLH gene, and the ectopic expression of which in *Arabidopsis* can improve their salt stress due to the increased germination rates and the emergence rates of green cotyledon [112]. In addition, bHLH TFs can also reduce harmful damage through the regulation of ROS activities when woody plants suffer from salt stress. In *Tamarix hispida*, ThbHLH1 improves salt tolerance by enhancing osmotic potential and decreasing reactive oxygen species accumulation, and the ectopic expression of *ThbHLH1* enhances the tolerance of salt in *Arabidopsis* [79]. *CfICE1*, a gene from *Cryptomeria fortune*, acts as a positive regulator in salt stress, and the ectopic expression of *CfICE1* in poplars improved their salt tolerance by increasing the antioxidative capacity and maintaining the structure of intracellular organelles [114]. Current studies have shown the roles of some bHLH TFs in woody plants when they encounter salt stress, but the regulatory mechanisms of bHLH TFs in woody plant responses still need to be further studied. Future research can be based on the existing theoretical results to excavate more bHLH genes that respond to salt stress in woody plants and carry out more in-depth practice in breeding.

#### 6.2.3. bHLH TFs Response to Cold Stress in Woody Plants

Low temperature is one of the main abiotic stress factors frequently encountered during plant growth, which affects the geographical distribution and growth of plants and reduces the yield and quality [141,142]. Continuous low temperatures will damage tissue structure and physiological function of woody plants [93,143]. Therefore, it is particularly important to explore the cold resistance mechanism of plants and further cultivate low-temperature-tolerant plant varieties. bHLH TFs families act as important regulatory roles in woody plants under low-temperature stress. For example, PavbHLH106 from sweet cherry is sensitive to cold, and the overexpression of *PavbHLH106* enhances the cold tolerance in transgenic tobacco [144]. *ICE1* is a kind of gene that encodes the MYC-like bHLH TFs and regulates the expression of the C-repeat Binding Factor (CBF), which plays a role in cold stress by activating genes involved in cold responses [145,146,147,148]. In apple, *MdCIbHLH1* (*Cold-Induced bHLH1*) encodes an ICE-like protein, which can improve cold tolerance in apple plants by upregulating the expression of *MdCBF2* [118]. Three *LcbHLH* genes in *Liriodendron chinense (Hemsl) Sarg.* have a positive trend in response to low-temperature stress, and LcbHLH24 may act as a positive regulator because its typical *ICE1* gene family structure [116]. In poplars, the ectopic expression of *CfICE1*, a bHLH gene from *Cryptomeria fortune*, improves cold tolerance by increasing the antioxidative capacity and maintaining the structure of intracellular organelles [114]. RmICE1 acts as a positive regulator of cold resistance in rose plants (*Rosa multiflora*) when they were under cold stress, and the overexpression of *RmICE1* in tobacco, enhancing their cold tolerance through increasing the activity of ROS scavenging and the expression of stress-responsive genes [115]. bHLH TFs in some woody plants enhance cold tolerance through oxidation-reduction system. In Chinese bayberry (*Myrica rubra*), MrbHLH10 played a positive role in cold stress through the modulation of ROS scavenging, and the transgenic *Arabidopsis* overexpressing *MrbHLH10* has higher APX activity and biomass accumulation in response to cold stress [108]. The ectopic expression of *PsbHLH42* from *Prunus sibirica* in *Populus ussuriensis* reduced the damage to membranes and increased the activities of SOD and POD in transgenic *Populus ussuriensis* under cold stress [48]. PubHLH1 in *Pyrus ussuriensis* played a positive role in low-temperature stress by maintaining a more effective detoxifying system and keeping slightly higher activities of CAT, POD, and SOD [117]. CsbHLH18 in sweet orange (*Citrus sinensis*) acts as a positive regulator in low-temperature stress through the regulation of antioxidant genes, thus improving the activities of antioxidant enzymes [119]. bHLH TFs in some woody plants enhance their cold tolerance not only through oxidation-reduction system but also through increasing the content of secondary metabolites. For example, *DlICE1* is an *ICE1*-like gene in *Dimocarpus longan* that encodes a protein with the bHLH domain, and the ectopic expression of *DlICE1* enhanced low-temperature tolerance in *Arabidopsis* by increasing the content of proline, decreasing the leakage of ions and reducing the accumulation of ROS [120]. The ectopic expression of *PtrbHLH* from *Poncirus trifoliata* in pummelo (*Citrus grandis*) enhanced low-temperature tolerance by reducing the accumulation of ROS, increasing the level of proline and improving the activities of antioxidant enzymes [121]. On the contrary, some bHLH TFs in woody plants play a negative role under cold stress. For example, the overexpression of *MdPIF3* in apples reduced cold tolerance in both apple callus and *Arabidopsis* plants [102]. At present, studies about the dual roles of bHLH TFs in cold stress are still not comprehensive enough, and more exploratory research needs to be carried out on the regulatory pathways of bHLH TFs in woody plants.

#### 6.2.4. bHLH TFs Response to Nutrition Deficiency Stress in Woody Plants

The content of various nutrient elements in the environment affects the growth and development of woody plants [149], and a lack of individual elements affect some basic physiological processes, including cell development, tissue differentiation and root growth of plants [150,151]. Therefore, it is particularly important to study the physiological network and molecular mechanisms of bHLH TFs in woody plants for various essential nutrients. Studies have demonstrated that bHLH TFs are widely involved in responses of iron deficiency stress. *PtFIT*, a bHLH TF gene from *Populus tremula*, was upregulated in roots under iron deficiency tolerance, indicating that it may play a positive role in iron-deficient stress [122]. A MxbHLH01 gene from the roots of *Malus xiaojinensis* was upregulated under iron-deficient conditions, and played a positive role in iron deficiency stress by forming heterodimers with other proteins [123]. MdbHLH104, a homolog of *Arabidopsis* bHLH104 in apple trees, acts as a key component in regulating plasma membrane H^+^-ATPase-mediated rhizosphere acidification and Fe uptake in apples (Malus domestica), which played a key role during Fe deficiency in plants [124]. MdbHLH104 can also directly bind to the promoter of *MdAHA8* to activate its expression and therefore enhance the ability of Fe uptake [152]. Systematic analysis and qRT-PCR showed that two bHLH TFs called CgbHLH16 and CgbHLH63 from pummelo (*Citrus grandis*) are possibly the key TFs that respond to iron-deficiency stress, and the protein–protein interaction prediction showed that *CgbHLH16* could interact with an important iron-deficiency responsive TF called PYE (bHLH47) [125]. *MxFIT* from *Malus xiaojinensis* is an iron-deficiency stress response gene which involved in the Fe uptake, and the ectopic expression of which improves the tolerance of iron-deficiency stress in *Arabidopsis* [126]. Nitrogen is also an essential nutrient for the growth of woody plants. Some bHLH TFs in woody plants play a negative role in nitrogen deficiency. For example, MhbHLH130 from apple (*Malus domestica*) played a negative role in nitrogen-deficiency stress by negatively regulating the expression of *MhCHS*, whose overexpression could increase the uptake of nitrogen [127]. When Poplar 84K (*Populus alba* × *P.tremula* var. *glandulosa*) was under low-nitrogen stress, 12 bHLH TFs were up-regulated, while 34 bHLH TFs were down-regulated, which indicate that these bHLH TFs might play important roles in the responses to low-nitrogen stress in Poplar 84K [128].

Increasingly, current research has focused on the functions of bHLH TFs in woody plants in response to nutrient element deficiency stress—a large number of bHLH TFs in woody plants have been gradually explored. For example, several bHLH TFs (bHLH1, bHLH30, bHLH81, bHLH72, bHLH45, and bHLH21) upregulated when *Neolamarckia cadamba* were encountered nitrogen deficiency [129]. In yellow horn (*Xanthoceras sorbifolia Bunge*), the gene MYC2-like, belonging to bHLH TFs, was up-regulated during nitrogen deficiency, which indicated this TF might be involved in the corresponding tolerance mechanism [130]. There is a growing body of evidence showing bHLH TFs playing active roles in other nutrition deficiencies in woody plants. MdSAT1, isolated from apples, was also a Pi (inorganic phosphate)-responsive bHLH transcription factor. The ectopic expression of *MdAST1* in *Arabidopsis* improved Pi deficiency tolerance through improving Pi utilization in response to a Pi deficiency, including increased lateral roots and root tips number and transcript levels of genes related to Pi uptake and transport [131]. Elucidating the response mechanisms and regulatory networks of bHLH TFs in response to nutrient deficiency stress in woody plants is of great significance for cultivating highly resistant varieties.

## 7. Conclusions and Perspectives

Plant responses to various environmental stresses are controlled by a cascade of molecular networks. Various stress-related molecules activate stress response mechanisms to re-establish cellular homeostasis and protect and repair damaged proteins and biofilms—the bHLH TF family plays a critical role in this process.

At present, many breakthroughs have been made in the study of the structure and function of bHLH TFs. However, physiological and biochemical studies on woody plants should be deepened to determine the function of bHLH TFs in the resistance responses to woody plants through genetic modification technology. In addition, studies on bHLH TFs families in woody plants are still relatively limited, and related studies are mostly limited to the model plants *Arabidopsis* and tobacco; thus, the functional specificity of bHLH TFs in woody plants still needs to be explored. Due to the diverse functions and complex mechanisms of bHLH TFs, it is still an important topic for further research in woody plants. In addition, unlike the single-gene traits, the responses to abiotic and biotic stresses of bHLH TFs in woody plants are the results of complex interactions of multiple genes, making it more difficult to design and control related experiments. Therefore, more effort should be made to systematically study the effects of multiple genes (especially regulatory genes) on woody plants. Importantly, improving the tolerance of woody plants to various stresses and breeding new varieties of woody plants with stronger tolerances will be the major directions for future studies on bHLH TF families.

## Figures and Tables

**Table 1:** bHLH TFs involved in the stress responses of woody plants.

Stress	bHLH TF	Species	Reference
Drought	MdPIF3	*Malus domestica*	[102]
	MdCIB1		[103]
	MdSAT1		[104]
	MdbHLH130		[92]
	PebHLH35	*Populus euphratica*	[105]
	PxbHLH02	*Populus simonii × P. nigra*	[106]
	ThbHLH1	*Tamarix hispida*	[79]
	HhbHLH2	*Hibiscus hamabo*	[107]
	MrbHLHp10	*Myrica rubra*	[108]
	PtrbHLH66	*Poncirus trifoliata* (L.) Raf.	[109]
	MabHLH144-like TFs	*Morus alba* L.	[110]
	CibHLH96	*citrus*	[111]
	SlbHLH	*Selaginella lepidophylla*	[112]
Increased Salinity	PsPRE1	*Populus simonii* ‘Tongliao1’	[113]
	MdSAT1	*Malus domestica*	[104]
	SlbHLHopt	*Selaginella lepidophylla*	[112]
	CfICE1	*Cryptomeria fortunei*	[114]
	ThbHLH1	*Tamarix hispida*	[79]
Low Temperature	MrbHLH10	*Myrica rubra*	[108]
	CfICE1	*Cryptomeria fortunei*	[114]
	RmICE1	*Rosa multiflora* Thunb.	[115]
	PsbHLH42	*Prunus sibirica*	[48]
	LcbHLH24	*Liriodendron chinense*	[116]
	PubHLH1	*Pyrus ussuriensis*	[117]
	MdCIbHLH1	*Malus domestica*	[118]
	CsbHLH18	*Citrus sinensis*	[119]
	DlICE1	*Dimocarpus longan*	[120]
	PtrbHLH	*Poncirus trifoliata*	[121]
Nutrient Deficiency	PtFIT	*Populus tremula*	[122]
	MxbHLH01	*Malus xiaojinensis*	[123]
	MdbHLH104	*Malus domestica*	[124]
	CgbHLH16,63	*Citrus Grandis*	[125]
	MxFIT	*Malus xiaojinensis*	[126]
	MhbHLH130	*Malus domestica*	[127]
	bHLH TFs	Poplar 84 K (*Populus alba* × *P. tremula var. glandulosa*)	[128]
	bHLH1,30,81,72,45,21	*Neolamarckia cadamba*	[129]
	bHLH (MYC2-like)	*Xanthoceras Sorbifolia Bunge*	[130]
	MdAST1	*Malus domestica*	[131]
Biotic Stress	PalbHLH1	*Populus alba var. pyramidalis*	[132]
	10 bHLH genes	*Prunus avium* L.	[133]
	bHLH TFs	*Juglans regia*	[134]

## Supplementary Materials

The following supporting information can be downloaded at: https://www.mdpi.com/article/10.3390/plants13162315/s1.

## Abbreviations

bHLH—basic helix–loop–helix; bHLHm1—bHLH membrane 1; TFs—transcription factors; LZ—leucine zipper; PIF—phytochrome interacting factor; ABA—abscisic acid; JA; SA—salicylic acid; ROS—reactive oxygen species; GR—glutathione reductase; CAT—catalase; POD—peroxidase; SOD—superoxide dismutase; GSH—glutathione; APX—ascorbate peroxidase; CBF—C-repeat Binding Factor.
