# Supplementary material for: Functions and Regulatory Mechanisms of bHLH Transcription Factors during the Responses to Biotic and Abiotic Stresses in Woody Plants
Source: Plants (Basel). 2024 Aug 20;13(16):2315. doi: 10.3390/plants13162315 (PMC11360703; doi:10.3390/plants13162315)
Supplement: Supplementary file 1 [file plants-13-02315-s001.zip › plants-3093080-supplementary.pdf]

Supplemental Table 1. The number of bHLH TFs and subfamilies identified in different species.

Species	Number of genes	Number of subfamilies	references
Arabidopsis	133	12	[45]
Nicotiana tabacum	100	15	[46]
Setaria Italica	187	23	[31]
Piper Nigrum	122	21	[47]
Prunus sibirica	104	23	[48]
Cyclocarya paliurus	159	26	[49]
Xanthoceras sorbifolia Bunge	136	26	[50]
Osmanthus fragrans	205	25	[51]
Prunus mume	100	21	[52]
Ginkgo biloba	85	17	[53]
Populus tremula	167	15	[54]
Populus deltoids	185	15	[55]
Rosa persica	142	21	[56]
